# Assessing the Impact of Interval Duration Between Ileal Pouch Creation and Loop Ileostomy Closure on the Development of Subsequent Inflammatory Pouch Conditions in Patients with Ulcerative Colitis

**DOI:** 10.1093/crocol/otaf005

**Published:** 2025-01-23

**Authors:** Mark Zemanek, Katherine Westbrook Cates, Joseph Carter Powers, Emma Dester, Qijun Yang, Riley Smith, Tracy Hull, Benjamin L Cohen, Taha Qazi

**Affiliations:** Department of Internal Medicine, Cleveland Clinic Foundation, Cleveland, OH, USA; Department of Internal Medicine, Cleveland Clinic Foundation, Cleveland, OH, USA; Cleveland Clinic Lerner College of Medicine of Case Western Reserve University, Cleveland, OH, USA; Cleveland Clinic Lerner College of Medicine of Case Western Reserve University, Cleveland, OH, USA; Department of Quantitative Health Sciences, Lerner Research Institute, Cleveland, OH, USA; Department of Internal Medicine, Cleveland Clinic Foundation, Cleveland, OH, USA; Digestive Disease and Surgery Institute, Cleveland Clinic, Cleveland, OH, USA; Digestive Disease and Surgery Institute, Cleveland Clinic, Cleveland, OH, USA; Digestive Disease and Surgery Institute, Cleveland Clinic, Cleveland, OH, USA

**Keywords:** Loop ileostomy, ileal pouch-anal anastomosis, pouchitis, ostomy duration

## Abstract

**Background:**

Many patients with medically refractory ulcerative colitis undergo ileal pouch-anal anastomosis, which typically includes the creation of a temporary loop ileostomy. The impact of the interval between ileal pouch-anal anastomosis and loop ileostomy closure regarding endoscopic pouch inflammation has not been well defined. The aim for this project was to assess if delayed loop ileostomy closure increases patients’ risk of endoscopic pouch inflammation.

**Methods:**

This is a cohort study of patients with ulcerative colitis who underwent ileal pouch-anal anastomosis between 01/2010 and 12/2020. Patients were divided into groups—early (12–116 days) or late closure (>180 days)—based on interval between ileal pouch-anal anastomosis and loop ileostomy closure. The late closure group was further sub-divided by indication for delay which included post-operative complications and non-medical reasons. The primary outcome was development of endoscopic inflammatory pouch disease, which was a composite of pouch disease activity index score of ≥ 4, mucosal breaks beyond anastomotic lines, and diffuse pouch inflammation.

**Results:**

Two-hundred ninety patients were included which comprised early and late cohorts of 217 and 73 patients, respectively. Compared to early closure, late closures for non-medical and pouch-related surgical complications were both not found to be associated with development of our composite outcome (*P* = .43 and *P* = .80, respectively).

**Conclusions:**

Delaying ileostomy closure due to patient preference or logistical limitations did not result in an increased risk of endoscopic pouch inflammation, but there appears to be an association of extraintestinal manifestations with endoscopic inflammatory pouch disease, suggesting the need for a vigilant surveillance in these patients.

## Graphical Abstract

**Graphical Abstract:**
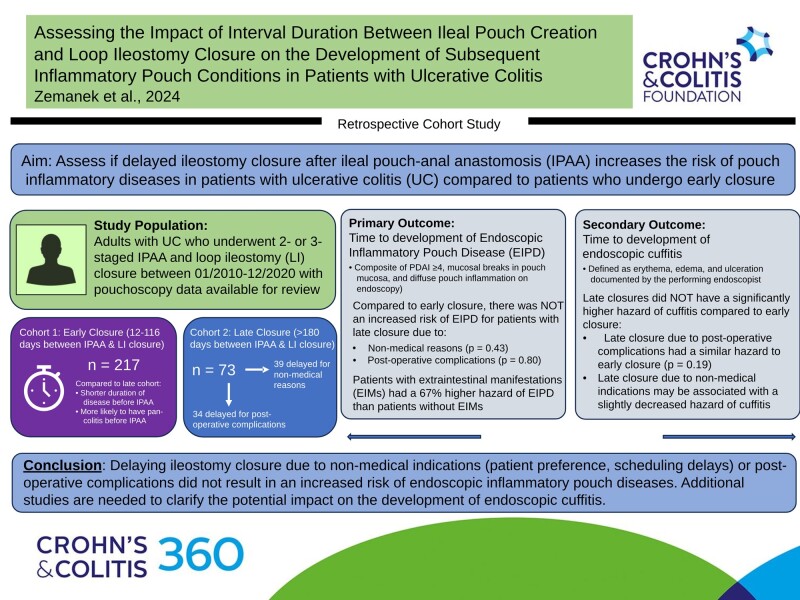

## Introduction

Ileal pouch-anal anastomosis (IPAA) is the surgical management strategy for patients with medically refractory ulcerative colitis (UC).^1^ The surgical approach is generally performed in multiple stages, including a total abdominal colectomy, creation of an ileal reservoir, and diverting loop ileostomy (LI), and finally ileostomy closure at a later date.^2^ A de-functioning LI is an important component of the IPAA surgical series as complications from anastomotic leak may be more prominent in patients without a diverting ileostomy.^3^ The interval between LI creation and closure may vary by institution and is impacted by medical and non-medical variables, but the typical interval is approximately three months. There is limited literature on variation in time intervals between IPAA and ileostomy closure in the context of subsequent pouch inflammatory complications including inflammation of the pouch (pouchitis) and cuff (cuffitis).^4^

The diversion of the fecal stream yields decreased nutrition received by the pouch which may foster a pro-inflammatory state. While most diverting LI are reversed, some situations may require a permanent ostomy. It is hypothesized that longer duration prior to ileostomy reversal results in a higher number of pouch complications due to prolonged starvation of fecal stream to the pouch.^5^ Studies have suggested that patients with delayed closure are at higher rates of complications.^6^ Given that >15% of patients with UC may require surgery by 10 years after diagnosis, early optimization of long-term pouch function by limiting inflammatory complications is crucial to decrease the risk of subsequent pouch failure.^1^ These complications include acute pouchitis, chronic pouchitis, and Crohn’s-like disease of the pouch (CLDP).^4^ It is estimated that 5-16% of UC patients that undergo IPAA develop subsequent CLDP, which significantly increases the risk of pouch failure and potentially permanent diversion ileostomy.^7^

Delays in ileostomy closure can be related to not only post-operative surgical complications but also non-medical factors such as patient preference and scheduling delays. Studies have suggested a higher rate of long-term complications in patients in the setting of post-operative complications, but inflammatory complications associated with delayed ileostomy closure due to patient preference or logistical considerations have not been established and thus represent a knowledge gap.^8^

The aim for this project was to assess if delayed ileostomy closure places patients at higher risk of subsequent endoscopic pouch or cuff inflammation. We evaluated patients separately for delayed closures secondary to medical/surgical complications and non-medical indications. We hypothesized that an increased time interval between IPAA and ileostomy closure is associated with inflammation of the pouch and is likely related to prolonged diversion of the fecal stream. Exploring this topic in greater detail will allow for a more informed decision regarding the optimal duration of time that the patient maintains a LI before returning to surgery for closure and subsequent risk stratification of inflammatory IPAA conditions.

## Methods

### Study Design

Our study is a retrospective cohort study that assessed patients with UC that underwent IPAA with LI closure between January 2010 and December 2020. Relevant inclusion criteria included age ≥ 18 years, a pre-operative diagnosis of UC, and a 2 or 3 stage IPAA surgery. Exclusion criteria for the study were a diagnosis other than UC leading to IPAA, colectomy prior to 2010 or after 2020, lack of pouchoscopy follow up ≥6 months following ileostomy closure, or age under 18 years old. Patients were divided into 2 groups—early (12–116 days) or late closure (≥ 180 days)—based on interval between IPAA and LI closure. The late closure group was further divided into post-operative complications and non-medical reasons as subtypes for the reason of delay. Information for each patient including demographics, surgical history, and UC clinical history, was collected from the electronic health record (EHR) and stored in a secure database. Extraintestinal manifestations (EIMs) including primary sclerosing cholangitis (PSC), pyoderma gangrenosum, arthralgias, aphthous stomatitis, psoriasis, uveitis/iritis/episcleritis, erythema nodosum, and ankylosing spondylitis were recorded when described before or after IPAA.

### Outcome Measures

The primary outcome was endoscopic inflammatory pouch diseases (EIPD), which was a binary composite of pouch disease activity index (PDAI)^8^ score of ≥4, mucosal breaks beyond anastomotic lines, and/or diffuse pouch inflammation. Patients who developed ≥1of the individual outcomes were deemed to have met the composite outcome. Secondary outcomes included endoscopic cuffitis and individual components of the primary outcome. Mucosal breaks included ulcerations, erosions, or aphthae noted on endoscopy that involved mucosa of the pouch body beyond anastomotic sites. Cuffitis was defined as description of inflammation (erythema, edema, ulcerations/mucosal breaks) on ≥1 endoscopic evaluation with or without symptoms. Diffuse pouch inflammation included erythema, induration, edema, or mucosal breaks noted in the pouch body, inlet, and afferent limb.

### Statistics

Data were described using median and interquartile range (IQR) for non-normal continuous variables and frequency (percentage) for categorical variables. The Pearson’s Chi-square test and Fisher’s exact test were used to compare the characteristics between two groups as appropriate. Univariate cox proportional hazards models were fit to test for association between individual variables and development of each outcome. A multivariable cox proportional hazards model was performed to control for potential confounders and variables identified as significant on univariate analysis. Restricted mean survival time (RMST) was reported for the specific late closure sub-group which violated proportional hazard assumption, and a stratified Cox proportional hazard model was applied to control the covariates which violated proportional hazard assumption. Analyses were performed using R software, and a significance level of 0.05 was assumed.

## Results:

### Patient Characteristics

**Figure 1.**
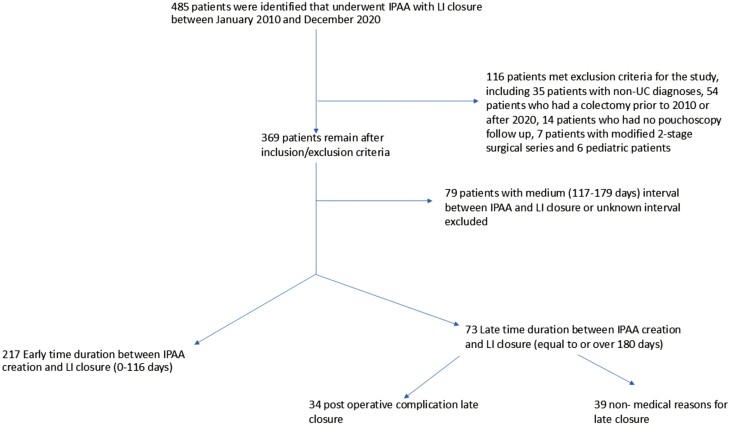
Patient inclusion/exclusion criteria by early vs. late time duration between IPAA creation and LI closure.

A total of 485 patients were identified that underwent IPAA between January 2010 and December 2020. From this group, 116 patients were excluded due to alternative diagnoses (ex: FAP, Crohn’s disease; 35 patients) leading to IPAA, colectomy occurring outside of the study interval (54 patients), lack of loop ileostomy (modified 2-stage surgical series; 7 patients), lack of pouchoscopy follow up (14 patients), or age <18 years at time of colectomy (6 patients). The interval between IPAA and ileostomy closure was calculated for each patient, and 79 patients with intermediate closure (117–179 days) were excluded from this study. There were 290 patients that remained and were included in the analysis (Figure 1). Demographics for the patient cohort are shown in Table 1. For this study, 1351 total pouchoscopies were reviewed with a median of 2 (IQR 1-4) pouchoscopies per patient. The median endoscopic follow-up times (until final pouchoscopy) after ileostomy closure for the early closure group was 26.3 months and 38.3 months for the late closure group. The median times to first pouchoscopy for the early and late closure cohorts were 11.6 months and 13.3 months, respectively. Dividing the late closure group, the median follow-up times post-ileostomy closure for the non-medical group was 40.2 months and 33.8 months for the post-operative complication group (Table 2).

**Table 1.** Summary of demographic variables by time between IPAA and loop ileostomy closure.

	[ALL]	Early	Late	*P*-value
	*N = 290*	*N = 217*	*N = 73*	*(Early vs late)*
Gender, *N* (%): Female Male	135 (46.6%) 155 (53.4%)	96 (44.2%) 121 (55.8%)	39 (53.4%) 34 (46.6%)	.220
Race, *N* (%):				.180
Asian	5 (1.72%)	4 (1.84%)	1 (1.37%)	
Black or African American	6 (2.07%)	6 (2.76%)	0 (0.00%)	
White	266 (91.7%)	200 (92.2%)	66 (90.4%)	
More than one race	8 (2.76%)	5 (2.30%)	3 (4.11%)	
Unknown/not reported	5 (1.72%)	2 (0.92%)	3 (4.11%)	
Age at time of colectomy, median [25th;75th]	36.8 [26.1;53.7]	36.6 [26.0;53.8]	37.7 [29.0;53.2]	.983
Duration of IBD Prior to Colectomy, *N* (%): <= 1 year >1 year Duration of IBD prior to colectomy (years), median [25th;75th]	25 (8.71%) 262 (91.3%) 5.85 [2.64;12.2]	23 (10.6%) 194 (89.4%) 5.44 [2.41;11.3]	2 (2.86%) 68 (97.1%) 7.28 [3.40;20.9]	.079 **.027**
Surgical indication, *N* (%):				.267
Medically refractory disease	231 (79.7%)	177 (81.6%)	54 (74.0%)	
Dysplasia/adenoma	43 (14.8%)	27 (12.4%)	16 (21.9%)	
Toxic megacolon	9 (3.10%)	7 (3.23%)	2 (2.74%)	
Other	7 (2.41%)	6 (2.76%)	1 (1.37%)	
BMI, *N* (%):				.067
Underweight	21 (7.27%)	19 (8.80%)	2 (2.74%)	
Normal Weight	138 (47.8%)	107 (49.5%)	31 (42.5%)	
Overweight	130 (45.0%)	90 (41.7%)	40 (54.8%)	
IBD distribution, *N* (%): Pan-colitis Left Sided Colitis/Proctitis	248 (86.4%) 39 (13.6%)	192 (89.7%) 22 (10.3%)	56 (76.7%) 17 (23.3%)	**.009**
Extraintestinal manifestations (ever), *N* (%): No Yes	168 (57.9%) 122 (42.1%)	123 (56.7%) 94 (43.3%)	45 (61.6%) 28 (38.4%)	.545
History of PSC (Ever), *N* (%): No Yes	272 (93.8%) 18 (6.21%)	203 (93.5%) 14 (6.45%)	69 (94.5%) 4 (5.48%)	1.000
Pre-operative Smoker?, *N* (%): No Yes	266 (91.7%) 24 (8.28%)	209 (96.3%) 8 (3.69%)	57 (78.1%) 16 (21.9%)	**<.001**
Backwash Ileitis, *N* (%): Yes No	56 (20.6%) 216 (79.4%)	45 (22.1%) 159 (77.9%)	11 (16.2%) 57 (83.8%)	.387
Number of stages, *N* (%):				**.003**
2	66 (22.8%)	39 (18.0%)	27 (37.0%)	
3	223 (76.9%)	178 (82.0%)	45 (61.6%)	
Surgical technique, *N* (%):				**.013**
Open	51 (17.7%)	30 (14.0%)	21 (28.8%)	
Laproscopic, converted to open	7 (2.43%)	7 (3.26%)	0 (0.00%)	
Pure laproscopic	229 (79.5%)	177 (82.3%)	52 (71.2%)	
Robotic Time from ileostomy closure to last pouchoscopy (months), median [25th,75th]	1 (0.35%) 31.4 [14.7;45.7]	1 (0.47%) 26.3 [13.8;45.1]	0 (0.00%) 38.3 [20.1;54.6]	**.007**
Post IPAA medications				
Oral corticosteroids	50 (23.0%)	23 (31.5%)	73 (25.2%)	.149
Topical corticosteroids	24 (11.1%)	3 (4.1%)	27 (9.3%)	.077
Immunomodulators	10 (4.6%)	4 (5.5%)	14 (4.8%)	.756
Small molecules	3 (1.4%)	1 (1.4%)	4 (1.4%)	1.000
Biologics	43 (19.8%)	17 (23.3%)	60 (20.7%)	.526
Oral 5-ASA	8 (3.7%)	4 (5.5%)	12 (4.1%)	.505
Topical 5-ASA	55 (25.3%)	17 (23.3%)	72 (24.8%)	.725
Antibiotics (overall)	154 (71.0%)	53 (72.6%)	207 (71.4%)	.789
Ciprofloxacin	106 (48.8%)	42 (57.5%)	148 (51.0%)	.199
Metronidazole	113 (52.1%)	39 (53.4%)	152 (52.4%)	.842
Tinidazole	35 (16.1%)	20 (27.4%)	55 (19.0%)	**.034**
Rifaximin	24 (11.1%)	11 (15.1%)	35 (12.1%)	.363
Vancomycin	28 (12.9%)	11 (15.1%)	39 (13.4%)	.639
Amoxicillin-Clavulanate	47 (21.7%)	19 (26.0%)	66 (22.8%)	.441
Trimethoprim-Sulfamethoxazole	17 (7.8%)	8 (11.0%)	25 (8.6%)	.411
Doxycycline	7 (3.2%)	2 (2.7%)	9 (3.1%)	1.000

**Table 2.** Summary of demographic variables of patients in the late closure by subgroup.

	[ALL]	Non medical	Post operative complication	*P*-value
	*N = 73*	*N = 39*	*N = 34*	
Sex (male), *N* (%):				0.875
Female	39(53.4%)	20 (51.3%)	19 (55.9%)	
Male	34 (46.6%)	19 (48.7%)	15 (44.1%)	
Race, *N* (%)				0.369
Asian	1 (1.37%)	0 (0.00%)	1 (2.94%)	
White	66 (90.4%)	34 (87.2%)	32 (94.1%)	
More than one race	3 (4.11%)	3 (7.69%)	0 (0.00%)	
Unknown / not reported	3 (4.11%)	2 (5.13%)	1 (2.94%)	
Age at time of colectomy, median [25th;75th]	37.7 [29.0;53.2]	32.8 [26.1;53.4]	38.4 [29.6;53.1]	0.744
Duration of IBD prior to colectomy, *N* (%):				1.000
<= 1 year	2 (2.86%)	1 (2.63%)	1 (3.12%)	
>1 year	68(97.1%)	37 (97.4%)	31 (96.9%)	
Duration of IBD prior to colectomy (years), median [25th;75th]	7.28 [3.40;20.9]	5.97 [3.40;16.2]	9.36 [3.58;23.1]	0.416
Surgical indication, *N* (%):				0.074
Medically refractory disease	54 (74.0%)	25 (64.1%)	29 (85.3%)	
Dysplasia/adenoma	16 (21.9%)	12 (30.8%)	4 (11.8%)	
Toxic megacolon	2 (2.74%)	1 (2.56%)	1 (2.94%)	
Other	1 (1.37%)	1 (2.56%)	0 (0.00%)	
BMI, *N* (%):				.103
Underweight	2 (2.74%)	0 (0.00%)	2 (5.88%)	
Normal weight	31 (42.5%)	20 (51.3%)	11 (32.4%)	
Overweight	40 (54.8%)	19 (48.7%)	21 (61.8%)	
IBD distribution, *N* (%):				.747
Pan-colitis	56 (76.7%)	1 (79.5%)	25 (73.5%)	
Left sided colitis/proctitis	17 (23.3%)	8 (20.5%)	9 (26.5%)	
Extraintestinal manifestations (ever diagnosed), *N* (%)				1.000
No	45 (61.6%)	24 (61.5%)	21 (61.8%)	
Yes	28 (38.4%)	15 (38.5%)	13 (38.2%)	
History of PSC (Ever), *N* (%):				.618
No	69 (94.5%)	36 (92.3%)	33 (97.1%)	
Yes	4 (5.48%)	3 (7.69%)	1 (2.94%)	
Pre-operative smoker? *N* (%):				.978
No	57 (78.1%)	31 (79.5%)	26 (76.5%)	
Yes	16 (21.9%)	8 (20.5%)	8 (23.5%)	
Backwash Ileitis, *N* (%)				.510
Yes	11 (16.2%)	7 (20.6%)	4 (11.8%)	
No	57 (83.8%)	27 (79.4%)	30 (88.2%)	
Number of Stages, *N* (%)				.713
2	27 (37.0%)	14 (35.9%)	13 (38.2%)	
3	45 (61.6%)	25 (64.1%)	20 (58.8%)	
Surgical technique, *N* (%):				1.000
Open	21 (28.8%)	11 (28.2%)	10 (29.4%)	
Pure laparoscopic	52 (71.2%)	28 (71.8%)	24 (70.6%)	
Time from ileostomy closure to last pouchoscopy (months), median [25th;75th]	38.3 [20.1;54.6]	40.2 [21.8;62.6]	33.8 [16.0;44.0]	.116

The majority of patients included were male (53.4%) and white (91.7%) and underwent surgical intervention for medically refractory disease (79.7%). The proportion of patients with a normal BMI (18.5-24.9) was 47.8%. Most patients were noted to have pancolitis distribution of disease (86.4%) prior to colectomy. The median age for patients at time of colectomy in this study was 36.8 (IQR 26.1–53.7) years. The median duration of UC prior to colectomy was 5.85 (IQR 2.64–12.2) years. Most patients underwent a pure laparoscopic (79.5%), 3 staged surgical series (76.9%). Extraintestinal manifestations (EIM) were common, with 122 patients (42.1%) demonstrating ≥1 EIM. Eighteen patients had a history of primary sclerosing cholangitis (6.21%).

Patients were divided into early and late closure cohorts based on the interval from IPAA creation until ileostomy closure. There were 217 patients in the early cohort and 73 patients in the late cohort. The late closure group had a slightly longer duration of IBD prior to colectomy and a lower percentage of patients with pancolitis disease distribution (Table 1), but the 2 groups were otherwise similar in terms of sex, age at colectomy, BMI, surgical indication, EIMs, history of PSC, surgical pathology, and surgical technique. Post-operative use of oral and topical corticosteroids, 5-aminosalicylates, immunomodulators, antibiotics (overall), and advanced therapies were not statistically significant between cohorts (*P* > .05 for all). Considering specific antibiotic therapies, the late closure cohort was slightly more likely to have used tinidazole (*n* = 20; 27.4%) compared to the early closure cohort (*n* = 35; 16.1%; *P* = .034), but there were no statistically significant differences regarding the use of ciprofloxacin, metronidazole, rifaximin, vancomycin, amoxicillin-clavulanate, trimethoprim-sulfamethoxazole, and doxycycline.

Of the patients in the late-closure group, 39 patients (53%) had delayed closure due to non-medical reasons or reasons unrelated to the IPAA, which consisted of patient preference or delays secondary to COVID pandemic re-scheduling of non-emergent procedures. The remainder of the late-closure group consisted of 34 patients (47%) who had delayed closure for post-operative complications consisting of sepsis, abscess formation, leaks, or fistula repairs (Table 2).

### Composite Outcome of Diffuse Pouch Inflammation, Mucosal Breaks Not Limited to Anastomotic Lines, PDAI > 4

**Figure 2.**
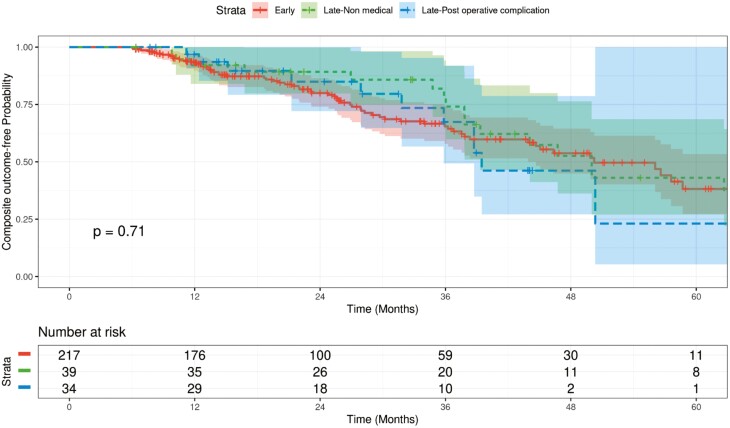
Kaplan–Meier curve for PDAI> = 4 or mucosal breaks or diffuse pouch inflammation: early vs. late (*N* = 290, event = 101).

There were 101 patients who met the criteria for the composite outcome (Figure 2). Of these 101 patients, 31 demonstrated the outcome on their first pouchoscopy, while the remaining 70 (69.3%) developed the outcome after the first pouchoscopy. Nineteen of the 31 patients had at least one subsequent pouchoscopy, from which 13/19 (68.4%) individuals demonstrated persistent disease (EIPD on at least one subsequent pouchoscopy). On univariable analysis, there was not a statistically significant difference in event-free survival for the early cohort and late cohort sub-groups (Figure 2).

Controlling for possible confounders, late closures for non-medical and pouch-related surgical complications were not found to be associated with development of our composite outcome (*P* = 0.43 and *P* = 0.80, respectively; Table 3). Of the included covariates, it was noted that EIMs were significantly associated with developing the outcome (HR = 1.67; 95% CI, 1.11-2.53; *P* = 0.014), with patients who have experienced ≥1 EIM throughout their disease course having a 67% higher risk of developing this outcome (Table 3).

**Table 3.** Multivariable cox proportional hazard model for development of the composite outcome in patients with early vs. late loop ileostomy closure (*N* = 282, event = 99)

Characteristic	HR^1^	95% CI^1^	*P*-value
Time between IPAA and ileostomy closure			
Early			
Late-non medical	0.81	0.47, 1.38	.43
Late-post operative complication	1.10	0.54, 2.22	.80
History of PSC (ever)			
No			
Yes	0.86	0.26, 2.85	.80
Extraintestinal manifestations (ever diagnosed)			
No			
Yes	1.67	1.11, 2.53	**.014**
IBD distribution			
Pan-colitis			
Left sided colitis/proctitis	0.99	0.50, 1.96	.97
Pre-surgery BMI			
Underweight/normal weight			
Overweight	0.70	0.45, 1.11	.13
Duration of IBD prior to colectomy (years)	0.98	0.95, 1.00	.10
Pre-operative smoker			
No			
Yes	1.58	0.79, 3.18	.20
Surgical technique			
Others			
Pure laparoscopic	0.93	0.57,1.51	.76
Number of stages			
2			
3	1.02	0.62, 1.68	.94

^1^CI = confidence interval; HR = hazard ratio.

### Cuffitis

There were 125 patients included in our study who developed endoscopic cuffitis. Of these individuals, 73/125 (58.4%) patients developed inflammation by the time of first pouchoscopy, while the remaining 52 (41.6%) demonstrated cuff inflammation only after the first pouchoscopy. Fifty-nine patients from this group had at least one subsequent pouchoscopy, from which 43/59 (72.9%) demonstrated persistent disease. The remaining 16 (27.1%) patients did not show evidence of cuff inflammation on subsequent pouchoscopies.

**Figure 3.**
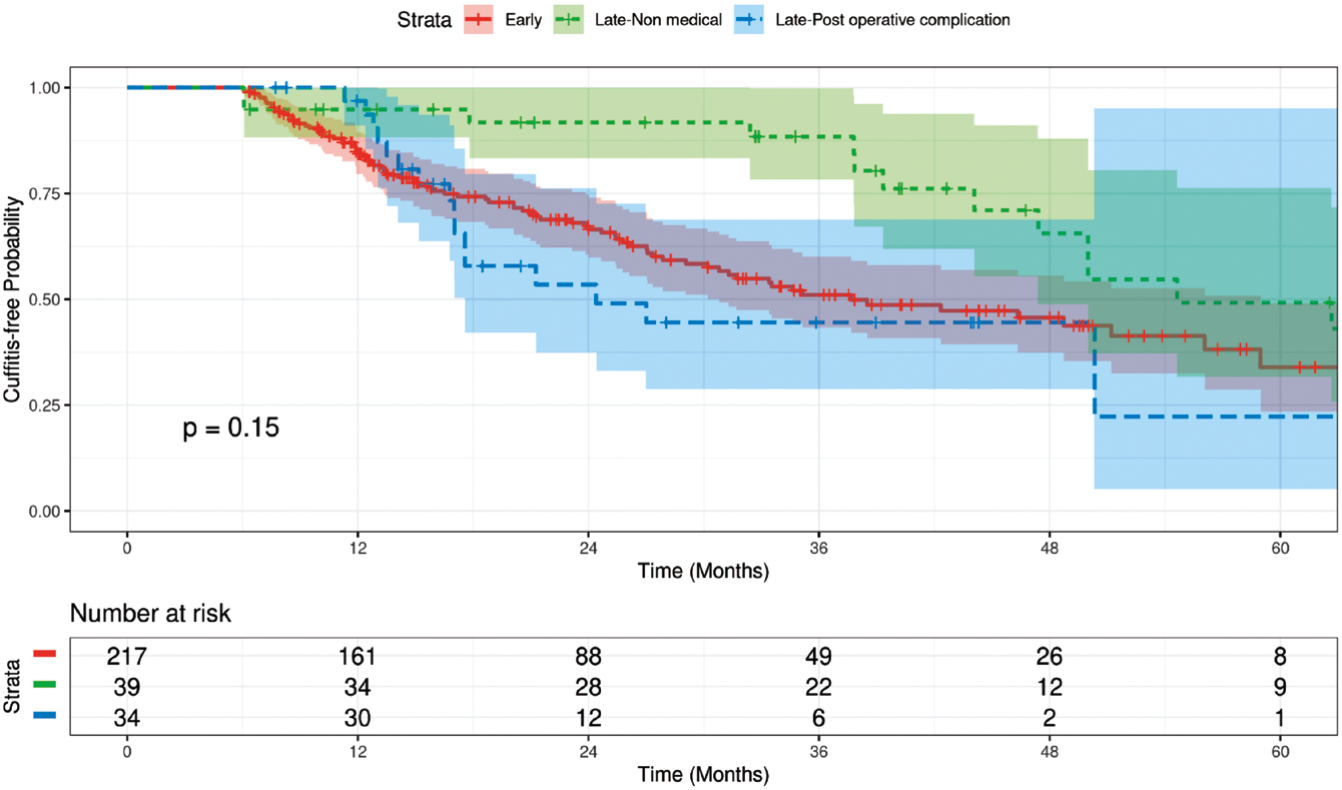
Kaplan–Meier curves for cuffitis: early vs. late (*N* = 290, event = 125).

The interval between IPAA and LI closure was not significantly associated with cuffitis development (Figure 3). It was noted that there was a violation of the proportional hazards assumption; therefore, a restricted mean survival/complication free time table was employed between the early and late closure subgroup who were delayed due to non-medical reasons. The RMST table showed that late closure due to non-medical reasons had a higher mean cuffitis-free time (Table 4) compared to the early group at 18, 24, 30, and 36 months.

**Table 4.** Restricted mean survival/complication free time (RMST) of early vs. late non-medical patients.

Follow-up period	RMST late (non medical) Estimate	RMST early estimate	RMST difference estimate (95%CI)	*P*-value
12 months	11.7	11.5	0.16 (−0.29, 0.60)	.493
18 months	17.4	16.2	1.16 (0.21, 2.11)	**.017**
24 months	22.9	20.5	2.40 (0.91, 3.94)	**.002**
30 months	28.4	24.2	4.22 (2.08, 6.35)	**<.001**
36 months	33.8	27.4	6.34 (3.56, 9.12)	**<.001**

For early versus late closure due to post-operative complications, our multivariable analysis showed no significant association of interval between IPAA and LI closure with cuffitis development (Table 5; HR = 1.49, 95 % CI, 0.82, 2.72, *P* = .19). However, it was noted that longer duration of IBD prior to colectomy was associated with a decreased hazard of the outcome in that every additional year of disease before one’s colectomy conveyed a 3% decreased hazard (HR = 0.97, 95% CI, 0.95, 1.00, *P* = .045).

**Table 5.** Multivariable cox proportional hazard model for cuffitis: early vs. late (stratified) (*N* = 244, event = 104).

Characteristic	HR^1^	95% CI^1^	*P*-value
Time between IPAA and loop ileostomy closure			
Early			
Late-post operative complication	1.49	0.82, 2.72	.19
Extraintestinal manifestations (ever diagnosed)			
No			
Yes	1.13	0.75, 1.71	.55
IBD distribution			
Pan-colitis			
Left sided colitis/proctitis	0.73	0.36, 1.48	.38
Pre-surgery BMI			
Underweight/normal weight			
Overweight	1.32	0.87, 1.98	.19
Duration of IBD prior to colectomy (years) pre-operative smoker	0.97	0.95, 1.00	**.045**
No			
Yes	0.82	0.32, 2.07	.67
Surgical technique			
Others			
Pure laparoscopic	1.23	0.72, 2.11	.44
Number of stages			
2			
3	1.29	0.76, 2.19	.35

^1^CI = confidence interval; HR = hazard ratio,

## Discussion

In this study, we assessed whether interval duration between IPAA and LI closure was associated with inflammatory conditions of the pouch. The colonic and enteric cells in the bowels are sustained by the fecal stream, and without the fecal stream, these cells are starved of nutrition.^9^ Due to this physiologic process, we chose to study whether delaying the ileostomy closure due to reasons that are not associated with medical complications portends a higher risk of subsequent pouch inflammatory complications. Particularly in UC patients, it is known that CLDP is a common complication, previously noted to be associated with late closure.^7^ However, fistulas and strictures were not evaluated in this study as the focus was specifically endoscopic inflammation of the pouch.

Ultimately, we did not find evidence to support an association of interval duration with the composite outcome, and thus we conclude that individuals who have late closure for non-medical reasons and pouch-related complications do not have significantly different risk of endoscopic pouch inflammation compared to patients who undergo early closure.

Of note, it is challenging to control confounding factors such as smoking status, disease duration, or type of procedure (ex: 3-stage group) between the early vs late groups. Other factors that are difficult to control may be related to the type of procedure that patients undergo. It was noted that patients undergoing 3-stage surgery are less likely to receive preoperative corticosteroid therapy in contrast to those undergoing 2-stage procedures.^2^ To address these potential confounders, we utilized multivariable models to control for potential effects attributed to variables other than interval duration, which was the primary focus of this study.

In contrast to the findings related to interval duration, EIMs were associated with an increased risk of primary outcome. This association is consistent with prior studies that showed EIMs may correlate with endoscopic disease activity of the pouch.^10,11^ EIMs are thought to foster a more complicated milieu that could potentiate late closure. Previous literature suggests that pouch inflammation is more common when an abnormal immune response is present, which could be why EIMs and autoimmune conditions, such as rheumatoid arthritis, have a higher risk of pouch inflammation.^12^

We also investigated whether cuffitis was associated with late closure due to non-medical reasons versus post-operative complications. For patients who had late closure due to non-medical reasons, a RMST table showed that late closure may actually have a protective effect based on this surgical approach in the development of subsequent cuffitis. In contrast, a multivariable model showed that late closure due to post-operative pouch complications had no significant association with cuffitis. This finding is significant as cuffitis has not previously been assessed in relation to the ileostomy closure interval. While more data is necessary to further characterize the relevance of cuffitis, the takeaway from our data suggests that late closure was not associated with an increased risk of the development of inflammatory pouch complications.

Previous literature suggests early closure of LI in patients with IBD who have undergone IPAA is associated with fewer pouch complications.^6,13^ Hyman et al concluded that there was no increased risk of pouch complications, specifically number of bowel movements and incontinence, from delay in ileostomy closure > 6 months.^14^

In a study that included patients from our institution, Vogel et al. reported surgical complications (specifically anastomotic leak, abscess formation, wound infection, ileus, or dehydration) to be significantly associated with very early closure, defined as ileostomy closure within 7-12 days from IPAA instead of the routine 2-4 months. Given the risks presented by this trial, it was stopped due to concern for patient harm.^15^ In another study from our institution, Clancy et al. did not note an increased risk of complications for patients with prolonged interval as a result of patient preference, but there was an increased risk of complications for patients who had a delay due to complications associated with IPAA.^16^ In times of early closures, the limited research in this topic has shown that closure within 56 days of LI does not convey additional risk of complications overall when compared to routine closure (56-116 days), but early closures specifically for stoma-related problems may carry a higher risk of complications as compared to early routine closures within 56 days.^17^

The aforementioned two studies suggested a difference in the subsequent development of pouch inflammatory complications in early versus later closure. This difference could be attributed to the earlier study assessing very early closure (prior to 56 days) compared to routine closure. While further ongoing studies are needed to differentiate this finding between early and late closure in previous literature, this may be significant in aiding patient decision making regarding the timing of ileostomy closure. To our knowledge, there has been no literature exploring cuffitis development relating to interval time. Therefore, further studies are warranted to contribute additional evidence to that presented in our study.

There is limited data on the role of post-operative complications and their association with subsequent pouch inflammatory complications. Although post-operative complications have been linked to fistula and stricture development, there is limited data on the long-term risk of subsequent inflammatory complications of the pouch. This is a knowledge gap that our study aimed to address. In our study, we included EIPD as a composite of PDAI score ≥ 4, mucosal breaks beyond anastomotic lines, and/or diffuse pouch inflammation. We found that while many patients develop inflammatory diseases by the time of first pouchoscopy, many also develop disesase in the more distant future following their ileostomy closure. Based on our analyses, we concluded that late closure was not associated with the development of EIPD.

There are significant clinical implications for this project as it suggests delaying ileostomy closure in the setting of patient preference (or scheduling delays as illustrated by the COVID pandemic) in the absence of medical or surgical complications does not place individuals at a higher risk of developing inflammatory pouch complications.

Our study also adds that EIMs are associated with the subsequent development of pouch inflammatory complications. EIMs are likely indicative of a higher risk of inflammatory pouch disease likely due to an increased inflammatory state in the body. Therefore, in patients with a history of EIMs, providers should be cognizant of the risk of subsequent EIPD.

Our study has limitations that should be noted when reviewing the conclusions drawn from the work. The median patient age studied was 37 years old at a high-volume center which may impact the applicability of the findings to other clinical practices and hospitals. Given that this was a single-institution study, there are limitations regarding standardization of surgical approaches, endoscopic follow-up timelines, and reporting of pathologic findings. For example, a minority of patients in this analysis had a PDAI available at the time of endoscopy (9%) which may be more commonly utilized elsewhere. To address the subjectivity of individual endoscopists interpretation of pouchoscopies, objective findings including presence of mucosal breaks and site-specific inflammation were included for the analysis based on the established literature.^7,17,18^ Moreover, pouch and cuff inflammation were assessed through a direct review of pouch endoscopic images by specialists and confirmed in the setting of disagreement by a gastroenterologist specializing in the management of inflammatory complications of the pouch.

An additional limitation for our study was present in assessing the cohort with late closure. The reason for delay was a concern that could skew the results of complications given the various reasons patients may necessitate a longer interval between surgeries, including the development of post-operative complications or medical complications, such as post-operative sepsis. To mitigate this limitation, we divided late closure into two subgroups, non-medical late closure and late closure due to post-operative pouch complications. This allowed us to better stratify our analyses and allow us to understand the risks of specific patient subgroups. In examining the lack of difference in the outcomes between the early and late ileostomy closure groups, there may be a correlation with overall clinical judgement for timing of the takedown. This may suggest that waiting longer for patients with more difficult pouch complications or challenging patients may actually result in more successful outcomes.

A final limitation from our current study design relates to the exclusion of the intermediate closure cohort from the statistical analysis. This approach was chosen in order to compare distinct populations of individuals (early vs. late), which would not have been as apparent with comparisons made to the intermediate group which included individuals similar to eight of the two extreme cohorts. As an alternative approach, incorporating “time to ileostomy closure” as a continuous predictor variable could have been considered in place of utilizing cohorts of individuals. This approach would have allowed all individuals meeting eligibility criteria to be included, but the interpretability and clinical utility of a continuous predictor for this study would have been less than using cohorts which can be easily compared. Furthermore, it is plausible that the variable may not demonstrate a linear response with the hazard of each outcome, which would make interpretation of the model results additionally complex. Therefore, the cohort based design was chosen for this investigation, but future studies may benefit from further exploring whether intermediate closures convey distinct risks compared to early and late closures.

## Conclusion

In summary, this study found that the timing of ileostomy closure did not portend the future development of endoscopic pouch inflammatory diseases. Specifically, delaying ileostomy closure due to patient preference or logistical limitations did not result in the development of endoscopic pouch inflammation, suggesting delays can be safe for this patient population. There is a potential protective role of delaying closure and the subsequent development of cuff inflammation, which needs to be further studied. There is an association of EIMs with EIPD, suggesting the need for a vigilant surveillance strategy in these patients. Future studies should follow patients for longer durations of time and incorporate additional objective measures of pouch health such as biomarkers to best understand these risks.

## Data Availability

Data not publicly available.
